# Age Estimation of Children and Young Adults of Jharkhand Using Mineralization of Third Molars and Its Relation to Chronological Age: A Retrospective Analysis

**DOI:** 10.7759/cureus.60431

**Published:** 2024-05-16

**Authors:** Swati Sharma, Nishita Garg, Prashant Gupta, Shantala R Naik, Sayani Roy, Abhishek Anand, Ajoy K Shahi

**Affiliations:** 1 Pedodontics and Preventive Dentistry, Dental Institute, Rajendra Institute of Medical Sciences, Ranchi, IND; 2 Oral Medicine and Radiology, Dental Institute, Rajendra Institute of Medical Sciences, Ranchi, IND; 3 Pedodontics and Preventive Dentistry, Netaji Subhas Medical College and Hospital, Bihta, IND; 4 Oral and Maxillofacial Surgery, Dental Institute, Rajendra Institute of Medical Sciences, Ranchi, IND

**Keywords:** mineralization, calcification, demirjian’s, impaction, third molar

## Abstract

Background

Dental age estimation plays an enormous role in the determination of an individual's identity and age in forensic and anthropological fields. The estimation of the chronological age of the individual is also important in the diagnosis, treatment planning, and treatment outcomes in the dental field. The third molar has some inimitable characteristics in terms of its size, shape, formation, and long path of eruption and usually erupts after puberty, which seems to be a reliable method of age estimation in adulthood. To establish the individual’s identity, inference of age has gained considerable attention in forensics, and the aspect of dentistry has broadened nowadays. Thus the present study was conducted.

Methodology

The digital orthopantomograms of 720 patients who were exposed to X-rays for routine examination were assessed, and calcification of the tooth was observed. In order to ensure the blinding of the examiners, radiographs were numerically coded. Clinical stages of the tooth were categorised into erupted, pre-erupted, and missing. Statistical analysis was performed by IBM SPSS Statistics for Windows, Version 25.0 (IBM Corp., Armonk, NY), with a level of significance set below 5%.

Results

The age of the patients whose OPGs were assessed ranged from 6 to 22 years, with a mean age of 18.93±3.129 years. Among the 720 participants, 370 (51.4%) were male and 350 (48.6%) were female. When the clinical status of the third molar among all the participants was assessed, in 148 (20.6%) subjects, third molars had erupted; in 188 (26.1%) subjects, the third molars were in the pre-erupted stage; and in 384 (53.30%), third molars were missing. When comparing the clinical status of the third molar in both arches and between genders, it was found that missing molars were common in females and the mandible arch, with statistically significant p-values. A comparison of Demirjian’s stages between genders showed that the mean age to attain stage H was 21.37±0.774 years among males and 21.69±0.616 years among females. This means that the calcification of third-molar attainment occurs earlier in males compared with females. In a similar comparison between the upper and lower arches, it was found that calcification of the third molar was attained earlier in the maxillary arch compared to the mandibular arch.

Conclusion

It was concluded that the third molar is a versatile tooth and its path of mineralization can be used in orthodontics, pedodontics, and forensics to estimate chronological age, and chronological age significantly follows Demirjian’s stages of third molar calcification. Third molar calcification occurred earlier in the maxillary arch and males, whereas several impacted molars were higher in females.

## Introduction

To establish an individual’s identity, inference of age has gained considerable attention in forensics, and the aspect of forensic dentistry has broadened nowadays [1]. Dental age estimation plays an enormous role in the determination of the individual’s identity and age in the forensic and anthropological fields [2,3]. The estimation of the chronological age is important for the attendance of schools and colleges, marriage, service, immigration, and criminal law cases [4].

The method of estimation of the chronological age is broadly classified into two categories: (i) based on the maturity of the bone and (ii) based on tooth development and eruption patterns. The estimation of chronological age through bone maturity is performed based on the radiological evaluation of certain bones, like fusion of long bone (epiphysis and diaphysis), medial edge of clavicle bone, epiphyseal top of the first rib, epiphyseal unification of the frontal iliac crest, and also the area where sphenoid bone fuses with the basal part of the occipital bone [5].

Estimation of the chronological age is generally made using radiographs. The most frequently used radiographs are orthopantomograms (OPGs) and cephalometric radiographs for estimating dental age [6]. The individual’s age in dentistry can be directly estimated based on clinical examination, which is based on the number of erupted teeth, sequence of eruption, and general condition, while the circumlocutory method of age estimation includes evaluation of stages of mineralization via intra- and extra-oral radiographs [2,3].

The third molar has some inimitable characteristics in terms of its size, shape, formation, and long path of eruption and usually erupts after puberty, which seems to be a reliable technique to estimate age in adulthood [7]. According to Martin-de las Heras et al., estimation of age is more difficult after 14 years because most of the teeth have completed their calcification and only the third molar is left for mineralization; thus, the third molar gains attention in age estimation [8]. From birth to maturity, dental age estimation has been changing, and the assessment of dental age in the younger age group is done by using the Moorrees et al. [9] chart and Gustafson's chart [10]. It has been observed that the growth, mineralization, and eruption of the third molar show a lot of variations. The initiation of growth usually starts at five to six years of age, and it reaches its peak around eight to nine years of age. At seven years of age, mineralization begins, and eruption of the third molar occurs after the formation of both enamel and root, which is usually finished around 18-25 years.

Therefore, after 14 years, the third molars are the only reliable source for estimating dental age. Age estimation via the third molar is based on the calcification stages proposed by Demirjian et al. In this chart, the correlation is performed between the mineralization stages of third molars on radiographic evaluation and the authentic age [11]. The radiographic evaluation of Dermirjian's stages is very useful in dental age assessment from 6 to 23 years [12]. As pubertal growth spurts and facial skeletal growth parameters are important pillars of diagnosis, treatment plan, and treatment outcomes in orthodontic as well as paediatric dentistry, the present study was conducted to estimate the age of children and young adults in Jharkhand based on the mineralization of mandibular third molars.

## Materials and methods

This retrospective study was conducted in the Department of Pedodontics and Preventive Dentistry in collaboration with Oral Medicine and Radiology from January 2021 to June 2022. In this study, the orthopantomograms (OPG) of 720 subjects were analysed.

The power analysis, which was performed using G*Power version 3.1.9.4, developed by Jochen Grommisch, demonstrated that with a sample size of 90 participants per group, it is possible to reach 85% power to identify significant differences at a 5% significance level. This estimate assumes an impact size of 0.342. The analysis setup includes eight groups and nine measurements per individual, with an assumed correlation of 0.5 between repeated measures. The results showed a noncentrality parameter (λ) of 18.9481680 and a critical F-value of 2.0548816. The numerator degrees of freedom were eight and the denominator degrees of freedom were 81. This configuration produces an actual power of 0.8618485. As a result, the total number of participants across all groups comes to 720, calculated as 90 subjects in each group.

Inclusion criteria and exclusion criteria

OPGs of 720 subjects between 6 and 22 years of age reporting to the Department of Pedodontics and Preventive Dentistry between January 2021 and June 2022 were included. Only those scans that displayed all third molars without distortions or deformities and free from any visible pathologies were included in the study. Scans that were lacking third molars, showing any form of obvious dental pathology, or exhibiting image deformity were excluded to ensure the accuracy and reliability of the mineralization assessments.

Data collection

This study was a double-blinded retrospective study. The study sample was divided into eight groups. Each group consisted of 90 subjects. The demographic details of the patient were obtained through a proforma. Orthopantomograms of 720 patients recommended for routine examinations were assessed, and calcification of the tooth was observed as per the methods outlined by Demirjian et al. [11]. Chronological age was computed by deducting the date of birth from the date of radiography for that specific person. To mitigate the effects of observer bias, each radiograph was assigned a numerical code. This ensured the examiners remained blinded to the gender and age of the individuals. To justify clinical stages of the tooth as erupted, pre-erupted, or missing, it was considered when more than two-third of the molars represented the same.

Written instructions, including drawings with written descriptions, were given to the evaluators of each group for Demirjian’s staging. Dermirjian’s staging scoring system contains 0 to 8 stages, with coding from A to H. For statistical computations, a numerical value was assigned to each stage, wherein stage 0 = 1, stage A = 2, stage B = 3, stage C = 4, stage D = 5, stage E = 6, stage F = 7, stage G = 8, and stage H = 9. Dermirjian’s Stages A-D depicted crown formation from the initiation of cusp calcification until the absolute crown formation, whereas Dermirjian’s Stages E-H depicted the formation of the root from the start of root bifurcation until the closure of the apex.

The scoring of the third molar was performed depending on the stage of calcification. Statistical analysis was performed by IBM SPSS Statistics for Windows, Version 25.0 (IBM Corp., Armonk, NY). A chi-square test was applied to quantify the association between clinical stages among arches and genders. A student ‘t’-test was applied to measure the relationship between the chronological age and the calcification stages of the subject's third molar teeth and to calculate the mean age to achieve Demirjian’s stages among males and females and between arches. For all analyses, the level of significance was set below 5%.

## Results

In total, 720 scans were evaluated for subjects whose age ranged from 6 to 22 years, with a mean age of 18.93±3.129 years. Of these, 370 (51.4%) were males and 350 (48.6%) were females. When the clinical status of the third molar among all participants was assessed, in 148 (20.6%) subjects, third molars were erupted; in 188 (26.1%) subjects, the third molars were in the pre-erupted stage and in 384 (53.30%), third molars were missing (Table 1). Of the missing 384 third molars, 159 were in the maxillary arch and 225 in the mandibular arch.

**Table 1:** Basic details of the study participants

Variables	Basic details	Frequency N (%)
Age	Mean	18.93 + 3.129
	Range	6–22 years
Gender	Male	370 (51.4%)
	Female	350 (48.6%)
Arch	Maxilla	290 (40.3%)
	Mandible	430 (59.7%)
Stage of eruption	Erupted	148 (20.6%)
	Pre-erupted	188 (26.1%)
	Missing	384 (53.30%)

**Table 2:** Comparison of the clinical status of the third molar in both arches and between genders P value < 0.05 considered significant

	Clinical status			Chi-square value	P value
	Erupted	Pre-erupted	Missing		
Maxilla	78 (9.7%)	61 (11.4%)	159 (20.6%)	8.028	0.018
Mandible	70 (2.9%)	127 (18.6%)	225 (36.5%)		
Total	148 (12.7%)	188 (30.1%)	384 (57.1%)		
Male	98 (8.5%)	126 (15.5%)	146 (25.3%)	58.413	0.001
Female	50 (4.1%)	62 (14.5%)	238 (31.8%)		
Total	148 (12.7%)	188 (30.1%)	384 (57.1%)		

Missing molars were more prevalent in the mandible when the clinical state of the third molar in both arches was compared in this study (p = 0.018). The study observation also indicated that missing third molars were more common in females (p = 0.001), as seen in Table 2.

Demirjian’s Stages A to H were determined as shown in Figures 1-8. 

**Figure 1:**
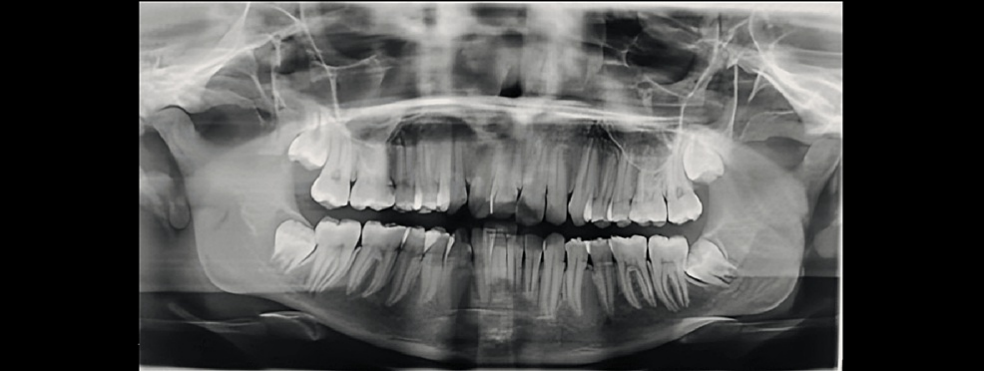
Orthopantomograph of a female child aged 14 years. Tooth germs of maxillary and mandibular third molars are visible (showing Demirjian formation stage F)

**Figure 2:**
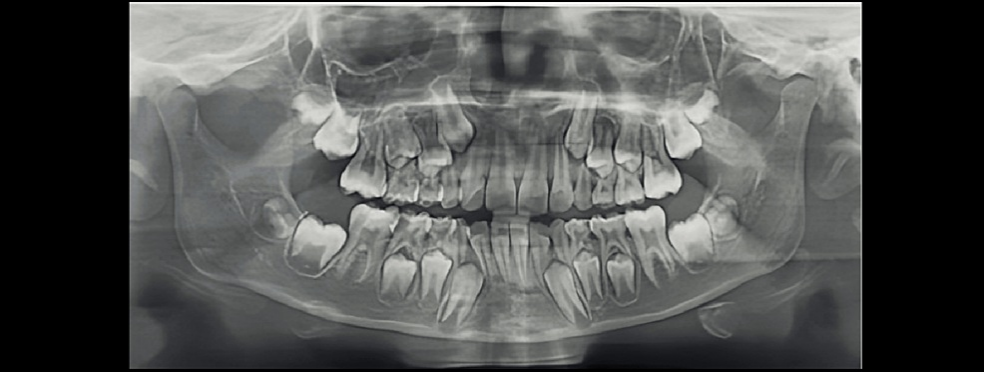
Orthopantomograph of a male child aged eight years. Tooth germs of maxillary and mandibular third molars are visible (showing Demirjian formation stage B)

**Figure 3:**
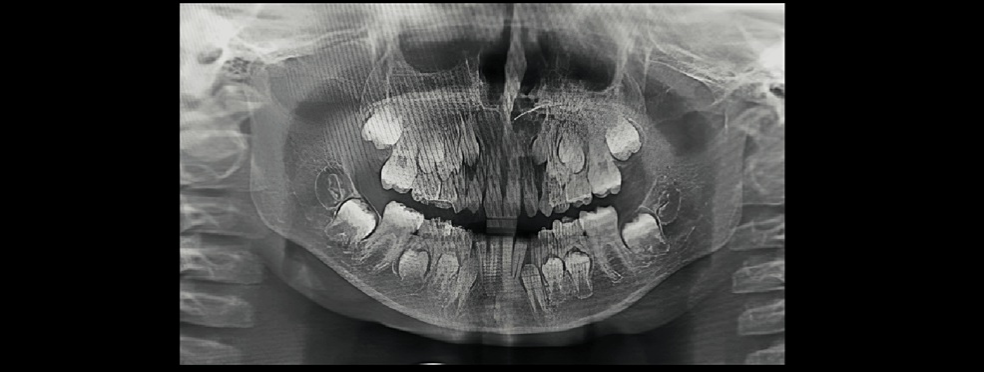
Orthopantomograph of a male child aged seven years. Tooth germs of mandibular third molars are visible (showing Demirjian formation stage A)

**Figure 4:**
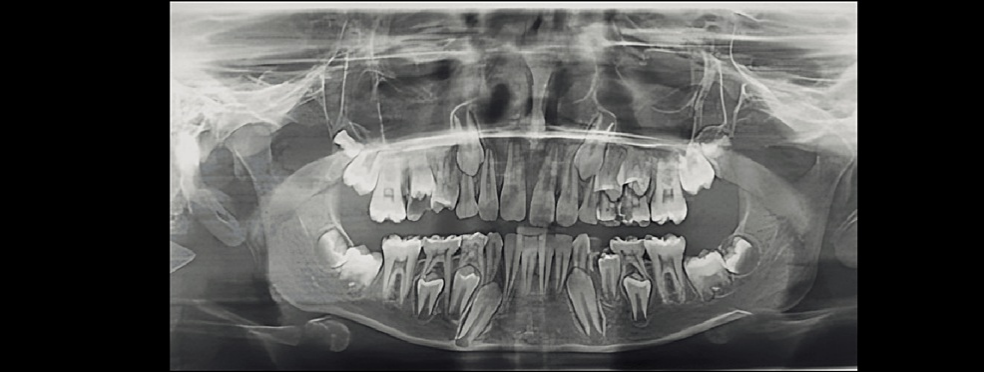
Orthopantomograph of a male child aged 12 years. Tooth germs of maxillary and mandibular third molars are visible (showing Demirjian formation stage C)

**Figure 5:**
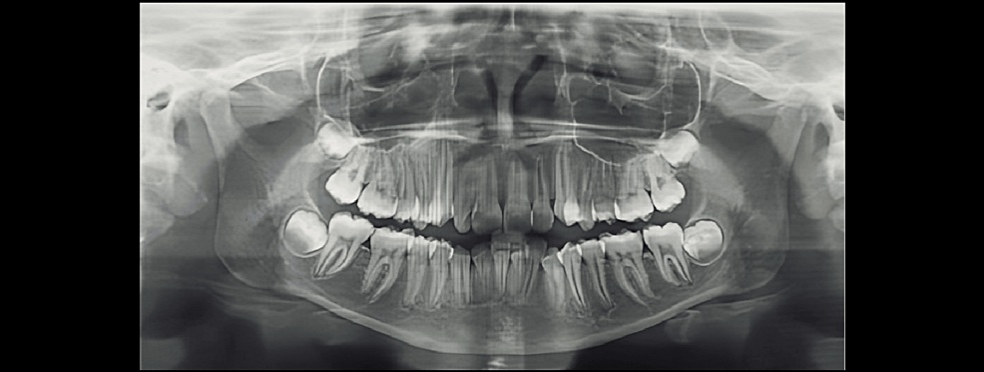
Orthopantomograph of a female child aged 13 years. Tooth germs of maxillary and mandibular third molars are visible (showing Demirjian formation stage D)

**Figure 6:**
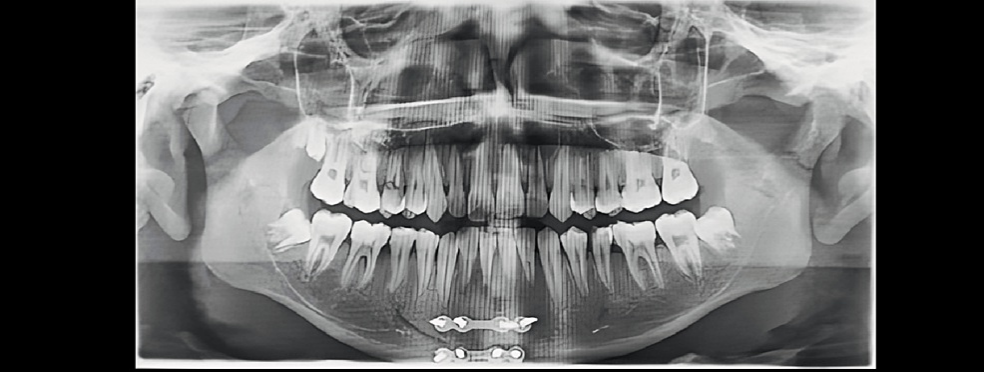
Orthopantomograph of a female child aged 15 years. Tooth germs of maxillary and mandibular third molars are visible (showing Demirjian formation stage E)

**Figure 7:**
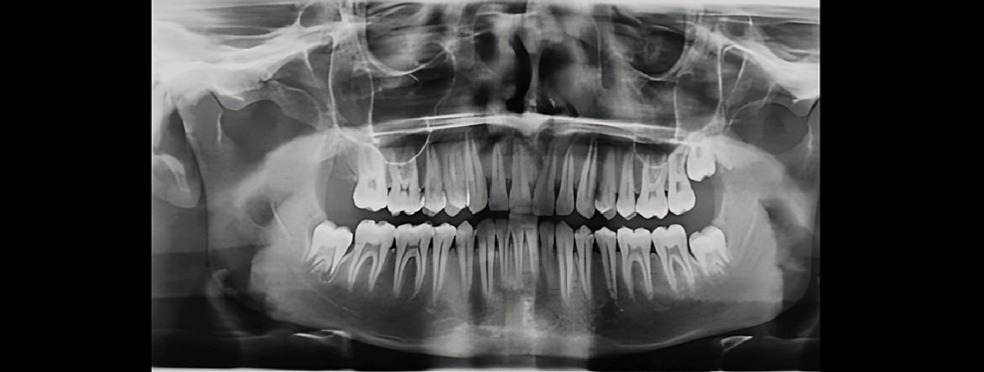
Orthopantomograph of a male child aged 16 years. Tooth germs of maxillary and mandibular third molars are visible (showing Demirjian formation stage G)

**Figure 8:**
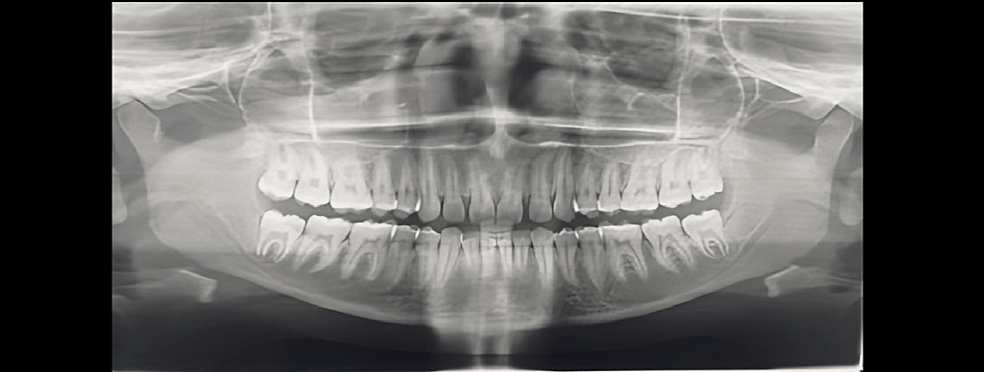
Orthopantomograph of an adult female aged 19. Tooth germ of maxillary and mandibular third molars are visible (showing Demirjian formation stage H)

On comparison, it was seen that the mean age to reach stage A was 6.84±0.723 years in males and 7.16±0.547 years in females (p = 0.041). The mean age to reach stage B in males was 9.29±0.602 years and 9.52±0.505 years in females, but the difference was not significant, while the mean age to attain stage C was 12.76±0.989 years in males and 13.93±0.728 years in females, with a highly significant p-value of 0.001. The mean age to reach stage D in males was 19.00±1.184 years and 19.56±1.196 years in females, with a statistically significant p-value of 0.02. The mean age to reach Demirjian’s Stage E in males was 19.40±0.494 years and 19.63±0.486 years in females (p-value = 0.001). The mean age to reach Demirjian’s Stage F was 19.36±0.775 years in males and 20.26±1.109 years in females, with a p-value of 0.001. For reaching Demirjian’s Stage G, the mean age was 20.45±0.812 years in males and 20.70±0.814 years in females (p = 0.037). Whereas males reach stage H at the mean age of 21.37±0.774 years, females reach this stage at the mean age of 21.69±0.616 years (p = 0.026). It can thus be observed that calcification of the third molar was achieved earlier in males compared to females (Table 3).

**Table 3:** Comparison of Demirjian’s Stages among males and females *=Significant; NS = Not Significant

	Male		Female		T-test value	P value
	Number	Mean±SD	Number	Mean±SD		
Stage A	32	6.84±0.723	38	7.16±0.547	-2.068	0.042*
Stage B	41	9.29±0.602	43	9.52±0.505	-1.896	0.061 (NS)
Stage C	59	12.76±0.989	57	13.93±0.728	-7.219	0.001*
Stage D	60	19.00±1.184	56	19.56±1.196	-3.193	0.02*
Stage E	50	19.40±0.494	47	19.63±0.486	-2.608	0.01*
Stage F	48	19.36±0.775	42	20.26±1.109	-5.204	0.001*
Stage G	45	20.45±0.812	36	20.70±0.814	-2.099	0.037*
Stage H	35	21.37±0.774	31	21.69±0.616	-2.265	0.026*

In the present study, Demirjian’s Stages A to H were determined, and on comparison, it was noted that the mean age to reach stages A to C in the maxillary arch was 6.78±0.441, 8.83±0.389, and 13.10±1.021 years, respectively, while in the mandibular arch, it was 7.47±0.507, 9.58±0.499, and 13.93±0.710 years, respectively (p = 0.001). The mean age to reach stage D was 18.79±1.041 years in the maxillary arch and 20.09±1.083 years in the mandibular arch; the difference was statistically significant (p =0.01). The mean age to reach Demirjian’s Stage E in the maxillary arch was 19.22±0.418 years and in the mandibular arch, it was 19.79±0.549 years (p = 0.001). The mean age to reach Demirjian’s Stage F in the maxillary arch was 19.68±0.755 years and in the mandibular arch, it was 20.02±1.035 years, with no statistical significance. The mean age for Demirjian's Stage G to appear in maxillary and mandibular arches was 20.44±0.796 and 21.21±0.650 years, respectively (p = 0.001), whereas stage H was achieved in the maxillary arch at the mean age of 21.23±0.805 years and in the mandibular arch at the mean age of 21.74±0.561 years (p = 0.0039). It can be seen that the calcification of the third molar was attained earlier in the maxillary arch compared to the mandibular arch (Table 4).

**Table 4:** Comparison of Demirjian’s Stages among maxillary and mandibular arches *=Significant; NS=Not Significant

	Maxilla		Mandible		T test value	P value
	Number	Mean±SD	Number	Mean±SD		
Stage A	09	6.78±0.441	32	7.47±0.507	-3.706	0.001*
Stage B	12	8.83±0.389	52	9.58±0.499	-4.825	0.001*
Stage C	20	13.10±1.021	60	13.93±0.710	-4.050	0.001*
Stage D	71	18.79±1.041	80	19.79±0.549	-6.453	0.001*
Stage E	52	19.22±0.418	71	20.02±1.083	-5.743	0.001*
Stage F	47	19.68±0.755	52	20.09±1.035	-1.804	0.075 (NS)
Stage G	48	20.44±0.796	48	21.21±0.650	-4.625	0.001*
Stage H	31	21.23±0.805	35	21.74±0.561	-3.057	0.003*

## Discussion

The present study was conducted to estimate the age of children and young adults based on the mineralization of mandibular third molars. The study was conducted among 720 subjects in the age range of 6 to 22 years, with a mean age of 18.93±3.129 years. Among the 720 participants, 370 (51.4%) were male and 350 (48.6%) were female. A similar study was conducted among 848 South Indians, of whom 55.4% were male and 44.5% were female, aged between 14 and 30 years [13].

On evaluating the clinical status of the third molar, 148 (20.6%) subjects had fully erupted third molars, 188 (26.1%) were in the pre-erupted stage, and 384 (53.30%) were missing the third molars. The missing teeth were commonly found in the mandibular arch, and erupted teeth were more common in the maxillary arch. Another study conducted in the Indian context also reported that mandibular third molars were missing [13]. Similarly, Pillai et al. [14] also reported that the prototype of the third molar impaction was more common in the mandible arch. This finding could be attributed to several factors, such as the fact that the maxillary bone generally provides more space for eruption than the denser, more constrained mandibular bone, thus allowing a smoother eruption process. The root formation of the latter often promotes easier eruption because of favourable development and angulation. In addition, genetic predispositions and environmental influences, such as diet and overall health, might play a significant role in influencing eruption patterns differently [14]. In this study, when the clinical status of the third molar was compared between genders, it was found that missing teeth were more common in females, whereas erupted teeth were more common in males. Similar results were reported in the study of Sivaramakrishnan and Ramani [15]. The mandibular arch has a higher predisposition to impacted third molars, and females have a higher predilection to impacted third molars. Passi et al. found that males (60.8%) were more likely to present with impacted mandibular third molars than females (39.2%) [16].

Over a long period, tooth development can be used to estimate chronological age and calcification of the third molar can be utilized as a powerful tool to assess age [17,18]. Demirjian’s stages method was utilised to estimate the calcification of the third molar, and a comparison was made between chronological age and dental age on radiographic evaluation of stages A to H. In this study, males reach Demirjian’s stage H at the mean age of 21.37±0.774 years and females at the mean age of 21.69±0.616 years, with a significant difference. Similarly, Priyadharshini et al. [13] found that on radiological investigation, the males in a sample of Chennai population reached stage H at a mean age of 22.88 years, whereas females did it in 23.35 years. While according to other studies among the Turkish population, the mean age to attain stage H was around 20.1 years, 22.1 years in males, and 22.6 years in females [7, 17]. As per the present study, it was found that the mean age to achieve Demirjian’s stages among males is earlier compared with females; it was observed that calcification of the third molar is attained earlier in males compared with females. A similar result was found by Priyadharshini et al. [13], while in contrast to our study, some other studies reported that the mean age to attain Demirjian’s stages was lower in males as compared to females [17,19]. These differences may be due to the disparity in the selected age groups in different studies and differences in genetic, environmental, and growth factors [20].

In the present study, it was found that calcification of the third molar occurs earlier in the maxillary arch as compared to the mandibular arch. Similarly, according to Priyadharshini et al., a significant difference was observed in the calcification stages F and G of the third molar in maxillary and mandibular arches, and the eruption of the maxillary third molar attained Demirjian formation stages in males [13]. Jung et al. also reported that the growth and maturation of third molars were attained earlier in the upper arch and males as compared to the lower arch and females [21]. In humans, the most versatile tooth in terms of size, shape, and pattern is the third molar, which exhibits a long path of formation. The calcification of the third molar is usually completed following the onset of puberty, as in this period, very few dental skeletal markers are available to estimate the age; therefore, the third molar gains more attention in orthodontics and forensic sciences. In this study, it was found that chronological age can be identified by the calcification or mineralization of the third molar in a sample population of Jharkhand, and radiological investigation of Demirjian’s stages acts as a potent tool to correlate calcification stages with chronological age.

Certain limitations of the study need to be mentioned. First, the accuracy of third molar mineralization as an indicator of chronological age can vary significantly among different populations due to genetic, dietary, and environmental factors, which may not be fully accounted for in a single region like Jharkhand. Additionally, the retrospective design of the study might introduce biases related to the selection of study subjects and the accuracy of recorded ages. There may also be limitations in the imaging techniques used to assess molar development; differences in resolution and interpretation can affect the precision of age estimates.

## Conclusions

The present study concluded that the third molar is a versatile tooth and its path of mineralization can be used in orthodontics and forensics to estimate chronological age, and chronological age is significantly related to Demirjian’s stages of third molar calcification. Males acquired calcification of the third molar earlier compared with females, but females have more impacted third molars. Nowadays, the third molar is gaining more attention in forensics and orthodontics planning due to its long course of formation and as a good dental skeletal marker.
